# Surveying immigrants without sampling frames – evaluating the success of alternative field methods

**DOI:** 10.1186/s40878-016-0044-9

**Published:** 2017-01-03

**Authors:** David Reichel, Laura Morales

**Affiliations:** 1 European Union Agency for Fundamental Rights, Vienna, Austria; 2 University of Leicester, Leicester, UK

**Keywords:** Survey, Sampling, Immigrants, Hard-to-reach groups

## Abstract

This paper evaluates the sampling methods of an international survey, the Immigrant Citizens Survey, which aimed at surveying immigrants from outside the European Union (EU) in 15 cities in seven EU countries. In five countries, no sample frame was available for the target population. Consequently, alternative ways to obtain a representative sample had to be found. In three countries ‘location sampling’ was employed, while in two countries traditional methods were used with adaptations to reach the target population. The paper assesses the main methodological challenges of carrying out a survey among a group of immigrants for whom no sampling frame exists. The samples of the survey in these five countries are compared to results of official statistics in order to assess the accuracy of the samples obtained through the different sampling methods. It can be shown that alternative sampling methods can provide meaningful results in terms of core demographic characteristics although some estimates differ to some extent from the census results.

## Introduction

The importance of survey research has grown in the field of migration, particularly because migration and integration policies have become a policy priority in Europe in the past decade — at the local, regional, national and EU levels. The repeated call for sound data in the area of migration has motivated several targeted surveys among immigrant groups in the past decade (e.g. Beauchemin, Hamel, & Simon, 2016; Crul, Schneider, & Lelie, 2012; Ersanilli & Koopmans, 2011; Latcheva et al., 2006; Morales & Giugni, 2011; Recchi & Favell, 2009). Accompanying these data collection efforts, research on how to effectively collect information on immigrants through surveys has advanced as well (e.g. Beauchemin & González-Ferrer, 2011; Font & Mendez, 2013; Kraler & Reichel, 2010; Thomas, 2008). Recent studies have started looking into the main problems associated with surveying immigrants, primarily issues related to varying coverage and response rates of immigrant groups.

This paper evaluates a recent international sample survey, the Immigrant Citizens Survey (ICS), which aimed at surveying immigrants originating from countries outside the EU in 15 cities in seven EU countries (Huddleston & Tjaden, 2012). In five countries, Belgium, France, Hungary, Italy and Portugal, no suitable sample frame was available for the target population. Consequently, alternative ways to obtain a representative sample had to be found. In three of these (Hungary, Italy, and Portugal), the so-called ‘centres of aggregation sampling technique’ (Baio, Blangiardo, & Blangiardo, 2011) was employed, in France random dialling was employed, and in Belgium random routes including focused enumeration of the nearest neighbour technique was chosen as the sampling strategy. The main question driving this article is to what extent the ICS was able to obtain a representative sample of its target population, and what are the lessons learned for future surveys aiming at surveying immigrants, in particular, and hard-to-reach populations in general.

The paper assesses the main methodological challenges of carrying out a survey among a group of immigrants for whom no adequate sampling frame exists. We present the main methodological challenges of the survey and we provide an overview of the quality of the samples by analysing response rates. Furthermore, the samples of the survey in these five countries will be compared to results of official statistics in order to assess the likely accuracy of the samples obtained through the different sampling methods employed throughout the survey.

The article begins with background information about the survey, its aims, scope and target population. An outline of methods for sampling specific target populations is provided in the second section. We will not go much into technical detail of sampling techniques in this article, since a great body of literature has evolved in the past and the interested reader is referred to the relevant references. After that the third section describes the sampling methods used including the main challenges reported by the national survey coordinators and field workers. Section four presents findings related to the response rates in the different countries, and section five compares the sample to other data sources regarding the distribution of length of residence and the share of naturalised immigrants. The final section concludes the paper by making recommendations for the implementation of surveys dealing with similar challenges.

### Background: aim, scope and methodology of the ICS

The integration of immigrants, notably third country nationals (i.e. non-EU citizens, TCNs), is high on the policy agenda in Europe and there are several different data sources that provide information on the situation of immigrants in different social areas. Data on integration-related matters in the areas of employment, education, social inclusion and active citizenship have been published through Eurostat’s pilot study on indicators of immigrant integration (Eurostat, 2011), data on integration policies are available for instance from the Migrant Integration Policy Index (MIPEX; Huddleston, Niessen, Chaoimh, & White, 2011)^1^ and there are statistics available on the opinions of the general public toward the integration of immigrants (e.g. from the European Social Survey or Eurobarometer). Data on discrimination experiences and other data from selected groups of immigrants and ethnic minorities are collected by the European Union Agency for Fundamental Rights (FRA) through the European Union Minority and Discrimination Survey (EU-MIDIS) (FRA 2009a, Latcheva, Reichel, & Till-Tentschert, 2016) – with the second round having completed fieldwork in 2016.^2^

The main aim of the ICS was to ‘increase the voice of immigrants in the development of integration policies’ (Huddleston & Tjaden, 2012, p. 10) and therefore add immigrants’ subjective views and experiences to the debate on how well integration policies operate in Europe. The survey covered the themes of employment, language skills and training, political and civic participation, family reunion, long-term residence as well as naturalisation and citizenship acquisition of immigrants. According to the institutes who were responsible for managing the survey, the King Baudouin Foundation (KBF) and the Migration Policy Group (MPG), the specific objectives of the survey were to ‘increase the knowledge about migrants’ needs, experiences, aspirations – and of policy impacts[, to] assist policy actors in creating more effective integration policies and addressing the other factors that influence the integration process [and to] demonstrate the value of surveying immigrants for informing policies and public discourse’ (Huddleston & Tjaden, 2012, p. 11). The survey was carried out under the responsibility of the KBF and MPG, jointly with national coordinators and polling institutes, in 15 cities in seven European countries, including Belgium (Antwerp, Brussels and Liege), France (Lyon and Paris), Germany (Berlin and Stuttgart), Hungary (Budapest), Italy (Milan and Naples), Portugal (Faro, Lisbon and Setubal) and Spain (Barcelona and Madrid).

The definition of the target population included all persons who were not born in the country (i.e. first generation immigrants), who were non-EU citizens or stateless persons at the time of immigration (i.e. born as citizens of a country other than EU/EEA countries or Switzerland), residing in the country for more than 1 year (i.e. stable residence), holding or renewing a legal immigration status, and who were 15 years or older (Huddleston & Tjaden, 2012, p. 12). This means that the survey targeted long-term immigrants who are or have ever been subject to immigrant legislation as non-EU citizens. The survey therefore includes naturalised immigrants who previously held non-EU citizenship as well as non-EU citizens who were not born in the country of residence. Respondents might have had an illegal residence status at one point, but at the time of the survey they had to hold a legal residence status or be in the process of renewing an expired residence permit. In the following we abbreviate the target population as third country foreign-born persons (TCFB).

A requirement of the study was that all country partners aimed at probabilistic samples and should develop the best possible sampling strategy that is feasible in their country. This requirement led to the implementation of different sampling strategies, which are subject to inquiry in this paper. Before describing the sampling methods used, the next section offers a general overview of probability sampling and its application to difficult-to-reach subpopulations. We then move to present an analysis of issues related to non-response and a comparison of selected results of the survey with data from the census in order to assess the quality of the samples.

### Sampling methods and difficult-to-reach sub-populations

The use of population lists for sampling is considered a gold standard for obtaining representative probabilistic samples of a given target population. However, quite commonly, sampling from lists is not possible due to a variety of reasons that lead to the unavailability of adequate sampling frames. Sometimes registers of the resident population are inaccurate or outdated, which is especially true for migrant populations since migrants are more mobile than the non- or never-migrant population. Sometimes the necessary information for identifying a specific sub-population is not available from the existing registers (most notably the country of birth or nationality in the case of immigrants, cf. Kraler & Reichel, 2010). There are cases when it is known that existing registers are biased and, therefore, not useful for covering the target population. Lastly, population registers are sometimes simply not in place.

Given the need for reliable information on specific sub-groups, alternative methods of sampling allowing for the selection of probability samples were developed in the past decades. Table 1 provides a summarised overview of different sampling methods used to obtain probability samples that allow for the calculation of the probability of inclusion in the sample for each individual surveyed. The overview is not exhaustive but provides an indication of the range of available choices to obtain representative samples.^3^

**Table 1:** Examples of probability sampling methods for sub-populations in the absence of sampling frames

Suitable for (target population)	Type of sampling	Frames of random selection	Further reading (examples)
General population	Random digit dialling	Telephone numbers	-
General population	Random routes/random walk	List of households, place (time)	Brief overview described in FRA 2009b, p. 11–14
Sub-population/minor domains	Conventional household sampling with focused enumeration	List of households, place (time), clusters defined by selected addresses and adjacent households	E.g. Ipsos MORI, 2010; Erens, 2013, p. 52–53; or FRA, 2009b, p. 14–18
Sub-population/minor domains	Conventional sampling with Adaptive Cluster Sampling	List of households, place (time), clusters of neighbouring households	Verma, 2013
Sub-population/minor domains	Time-location sampling	Place (Time)	Baio et al., 2011
Sub-population/minor domains	Capture-Recapture	Place (Time)	Berry, 2007; Williams, 2010
Total or sub-population/minor domains	GPS based sampling	Place (GPS)	Landry & Shen, 2005
Sub-population/minor or mini domains	Respondent-Driven-Sampling (RDS)	Network	Heckathorn, 1997, http://www.respondentdrivensampling.org/
Sub-population/minor or mini domains	Snowball sampling	Network	Goodman, 1961
Sub-population/ethnic minorities/immigrants	Onomastic sampling	Names/telephone book	Schnell et al., 2013

If lists with good coverage are available, the likelihood of selection of each individual is known and simple random sampling (every individual has the same chance of being selected) or other random sampling variants (such as stratified or cluster sampling) can be used. In the absence of lists, common alternatives to produce probabilistic samples are random digit dialling, where random numbers are dialled and the persons answering the phone are deemed to be selected randomly (controlling for the number of phones a person has), and random routes, where interviewers are assigned a randomly-selected starting point and have to follow a specific route and select every n^th^ household. These sampling methods are commonly used for surveying the “general population” that lives in a given geographic area. However, for specific sub-populations these techniques usually do not pay off in terms of cost-effectiveness because the sampling process would take too long in order to find a large sample of the specific sub-groups. The smaller the size of the targeted sub-population, the less cost-effective these alternatives are, and immigrants are often a small sub-group in the overall population, especially once certain national origins are excluded from the target population.

Sub-populations or groups of persons within a population are usually called domains (Kalton, 2009, p. 125). Kish (1987, p. 36) differentiates between major, minor and mini-domains, representing ten or more percent of the total population, between 1 and 10% and between 0.1 and 1%, respectively. Sub-groups of less than 0.1% are termed ‘rare’ types. Minor and mini-domains require particular sampling methods as shown in Table 1.

Traditional sampling methods can be adapted and applied to sub-populations. For example, focused enumeration is a variant of random household sampling, by which every household selected through conventional sampling methods is asked if there are persons of the target population living in an adjacent household. This procedure has proven to work for minor domains and presents a booster sample just with an extended sampling frame but with only slightly higher screening costs. According to Erens (2013, p. 52–53) the method was developed in the 1980s by the National Centre for Social Research and the Policy Studies Institute (Brown & Ritchie, 1981 cited in Erens, 2013, p. 52). The method has been regularly applied in the UK and the United States for surveying ethnic minorities (Erens, 2013, p. 52–53). The EU-MIDIS I also used this technique in most EU member states to obtain a probability sample of specific ethnic groups (FRA, 2009b).

Another similar option is using Adaptive Cluster Sampling (ACS) to boost samples. ACS implies including all neighbouring sample units for each sampling unit that fulfils certain criteria (e.g. if households are sampled, the criterion could be that there is at least one member of the target group in the household). If a neighbouring unit also fulfils the criteria the (new) neighbouring units are selected as well. This continues as long as there are neighbouring units that fulfil the specified criteria. Hence, clusters of units meeting certain criteria that are neighbouring are all selected in the sample as long as one of the units in a cluster is randomly selected, and selection probabilities can be recalculated a posteriori. Based on the assumption (or knowledge) that specific target populations cluster in certain areas, it is efficient to select clusters of the target population, once the target group is found in a general population sample (Verma, 2013).

Random-digit-dialling (or sampling from lists of telephone numbers) can also be used for sub-populations by using screening questions at the beginning of each interview. If the target population is not too small, this method can also be applied efficiently. However, the costs are increased by the screening procedure if several questions are needed to identify the target population. Due to the high costs and inefficiency of screening via telephone sampling of the general population for some minor or mini domains, sometimes onomastic sampling is applied for ethnic minorities or immigrants (cf. Braun & Santacreu, 2009; Recchi & Favell, 2009). Onomastic sampling implies that the origin of persons is deduced from their names and/or surnames (cf. Schnell et al., 2013). Its use has been criticized due to the problems relating to the coverage of women who change names when they marry and to the complexities of onomastic identification, especially for the descendants of mixed marriages (cf. Brouard & Tiberj, 2011).

Alternative methods based on time-location sampling are also often used to sample sub-populations. These methods assume that it is possible to adequately reach and cover the target population at predefined locations through random sampling of those locations (at different times). One such method is the ‘centres of aggregation’ strategy, by which a list of non-residential locations where the target population concentrates – called centres of aggregation – is produced after extensive ethnographic research. Random selection from this list provides interviewers with the locations where they should, randomly as well, select final respondents. At the end of the interview, respondents are asked which of all places or locations included in the sampling list they usually visit. This information allows calculating the probability of inclusion in the sample at the selected locations. The method was further developed in Italy by the ISMU Foundation (see Baio et al., 2011 and Blangiardo, 2008) in order to sample immigrants, particularly undocumented immigrants, in the absence of comprehensive population registers. The challenge of this method is obviously to undertake adequately the ethnographic study that will find and list the universe of relevant locations, which fully cover the entire target population.

Somewhat related is the Capture-Recapture method, which samples a specific target population at a certain place and repeats the sampling at a later stage (cf. Berry, 2007). Through the calculation of the percentage of persons selected in both samples, the size of the target population can be estimated. This method can be used to estimate the size of the target population but not so much for creating a probability sample of sub-populations. Another method based on location sampling was employed to survey migrants in China through pre-defined geographical locations identified through GPS data. Locations were randomly sampled and interviews conducted, which gave better results than those obtained from the census (Landry & Shen, 2005). This method, again, is more appropriate to estimate the size of the target population, but not for creating a probability sample.

Completely different is the approach used by Snowball Samples or Respondent Driven Sampling (RDS), which rely on the network structure of a target population. It is assumed that all persons of the target population are linked through social contacts and therefore every member of the target population can (in theory) be reached through referral chains of other members of the target group. Selection through referral chains entails selecting first a number of individuals of the target population (through any of a multiplicity of possible methods), and subsequently selecting additional members of the target population who are identified through references made by the initial (or ‘seed’) respondents. At every subsequent stage every respondent is asked to refer to another person of the target population. Snowball sampling, which is today often considered a form of convenience sample, was originally developed for studying networks by Goodman (1961). RDS was specifically developed to makes inferences on certain hard-to-reach groups by Heckathorn and colleagues (see Heckathorn, 1997 and other references at www.respondentdrivensampling.org, accessed on 24 March 2016). RDS assumes that all members of a target population are linked through social contacts and therefore have a non-zero probability of being included in the sample if selected through chain referral methods. In addition, it assumes that the likelihood of selection of a respondent by an already selected respondent (or referee) is not influenced by whom the referee has been selected. For example, assuming that there are three individuals: A, B and C. A has named B and B has named C. The assumption is that the likelihood of selection of C is not influenced by A having selected B. This means that the selection propensities of respondents remain stable over several waves (i.e. a Markov Chain). If this assumption holds, the selection procedure converges in the end, which allows estimating the selection probabilities. RDS has been used for surveying immigrants, but the success of the method depends on several factors and assumptions, which are difficult to evaluate (Gile, Johnston, & Salganik, 2015). The method can also fail when an insufficient number of references to other members of the target population are made.

Both the snowball sampling and RDS are somewhat limited by the reliance on the assumption of full network connectivity. In some cases, this can be a ‘heroic’ assumption. In the case of its use for immigrant groups, while it is true that immigration often takes place through chain processes, the presence of multiple ‘isolates’ within the target population cannot be discarded. Certain types of migrants are not part of the chain-migration processes – for example, in some professional sectors, or those who arrive to the country of residence through family ties with non-members of the target population – and these individuals are more likely to have a zero (or close to zero) probability of selection if they do not mingle with other members of the target population. How relevant this exclusion is for the overall conclusions of any study will depend on the relative size of the ‘isolates’ within the target subpopulation and the substantive questions that the study aims at answering.

McKenzie and Mistiaen (2009) compared alternative methods for sampling immigrants including snowball sampling and a variant of the centre of aggregation method.^4^ Both methods show some sort of selectivity, where migrants who are more attached to their migrant community are over-represented. The over-representation of migrants with stronger links to the country of origin, is also shown by Beauchemin and González-Ferrer (2011), who evaluate the success of transnational snowball sampling in the context of African-European migrants. In this sense, the centre/location sampling appears to be more promising in that it can better approach a representative sample as compared to snowball sampling. Moreover, an additional drawback of snowball samples is that they sometimes require long chains, which are not easy to obtain. Given that snowball samples are on average more expensive than centre sampling, there seems to be a clear advantage of centre sampling compared to network sampling methods (McKenzie & Mistiaen, 2009).

**Fig. 1:**
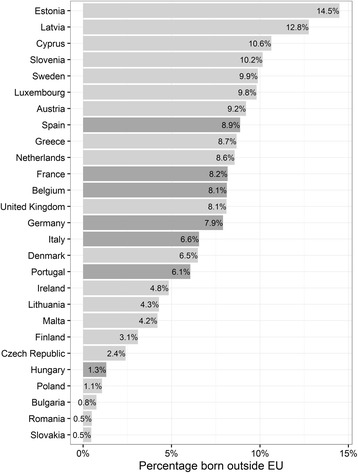
Distribution of the target population in EU countries in 2012 (except Croatia) – percent of immigrants from non-EU countries in the total population (*dark grey bars* indicate countries included in the ICS survey) Source: Eurostat database table ‘pop3-ctb’, extracted on 24 March 2016

To sum up, there is a variety of alternative methods to survey sub-populations, all of which have their strengths and weaknesses. The target population of the ICS can be defined as a minor domain in all countries included in that survey, as the target population represents between 1.3% of the total population (in Hungary) and 8.9% (in Spain) (see Fig. 1). These percentages are larger in the cities covered, as they are important destinations of immigrants within the selected countries. Moreover, alternatives to traditional random sampling methods were needed in most countries covered by the ICS, given that only two countries had adequate lists of their target population (Germany and Spain). The sampling methods used in each of the remaining five countries is described in the next section.

### Sampling strategies employed in the ICS

As mentioned above, convenience sampling – based on quotas or other criteria – was not an option for the survey, and this is the reason why alternative probabilistic sampling methods had to be employed. The decisions were taken by the national project teams in cooperation with their respective polling institutes. The following sampling strategies were chosen for the ICS after considering all possible options.

The Spanish samples were obtained through the nation-wide rolling local population register (*Padrón*), which provides a comprehensive register of the population, also including unauthorized immigrants (Duque, Ballano, & Perez, 2013, p. 70–71). However, the spatial distribution of the originally extracted simple random samples, which was deemed too dispersed territorially for the usual fieldwork standards of the polling institute, entailed that obtaining interviews would be too slow and fieldwork organisation would be much complicated. In order to reduce fieldwork costs, the sample was clustered by neighbourhood code and a new selection was done proportional to the size of the target population in the cluster, which transformed the sample into a clustered random one still allowing the calculation of selection probabilities. Consequently, designing a traditional random sample of individual TCFBs in the Spanish cities was not problematic, and the results are deemed representative of the target population aside from random sampling errors or any biases that might be introduced by non-response.

Germany is the second country where local population registers were used as a sampling frame. The situation in Germany turned out to be more complicated, however. Despite the fact that information on the country of birth of residents was in principle included in the German population registers, this information was not reliably entered into the local population registers in Berlin and Stuttgart and could not be used for sampling. Therefore, it was decided that the samples in the German cities will be based on nationality by selecting TCNs, who lived for more than 12 months in the city and had registered as having moved to the city from abroad (rather than another place in Germany). Hence, the operational definition of the target population in Germany deviates from the target population in the other countries to some extent, as it did not cover the real share of naturalised immigrants. In practice, it was found that there was still a considerable share of naturalised immigrants in the sample, whose change in citizenship was not recorded in the population register, which means that even nationality was not reliably captured in the German population registers.

In Belgium no adequate sampling frame for the target population was available, but information on the geographical distribution of third-country nationals by statistical wards (*Buurten/Quartiers*) was accessible for the year 2008, which was used as an indication of the densities of the target population. The number of TCFBs is almost certainly higher than the number of TCNs, because the former include naturalised immigrants as well.^5^ In order to make random selection feasible, a two-stage sampling design was employed. The Primary Sampling Units (PSUs) were the statistical wards. In the three cities a number of PSUs were selected proportional to the number of TCNs resident in the ward in 2008, once all PSUs with a percentage of TCNs below 10% were excluded. In PSUs with more than 18% TCNs traditional random route sampling was undertaken, whereas in PSUs with lower than 18% of the target population focused enumeration was employed (Ipsos, 2012). The exclusion of these PSUs was entirely based on cost-efficiency reasons: as it would be very costly and time-consuming to contact the target population in areas with low concentration. Undoubtedly, this introduces the potential for bias should the characteristics of the TCNs residing in areas of lower concentration of immigrants differ from those residing in areas of higher concentration.

Due to the absence of an adequate sampling frame and prior successful experience with similar surveys, a completely different approach was taken in France by sampling from telephone numbers. Telephone numbers were available for specific neighbourhoods, which were selected and stratified according to the number of immigrants recorded in the 2006 census, updated in 2009: neighbourhoods with less than 20% immigrants, with 20–40% immigrants and with 40 or more percent of immigrants. Phone numbers were randomly selected for each strata. The sample included 75% landlines and 25% mobile phone numbers. Individuals were screened through phone calls and after 6350 contacts a sample of 1004 (15.8%) was reached. A difference of the method in France is therefore that only telephone interviews were conducted compared to face-to-face interviews in all other countries.^6^

The remaining three countries, Italy, Hungary and Portugal, opted for location sampling (Centres of Aggregation Technique), which entails selecting a number of locations where immigrants are likely to be found. Sampling was carried out at those locations (e.g. public places, ethnic shops and restaurants, counselling organisations, service centres, etc.) and weights of inclusion probabilities were calculated according to the respondents’ attendance profile. It was also possible to include sampling from population registers as one of the locations/centres covered, something that was done in Hungary. This means that a random sample was taken from the public registry and respondents were asked if they also attend other centres included in the sample.

**Fig. 2:**
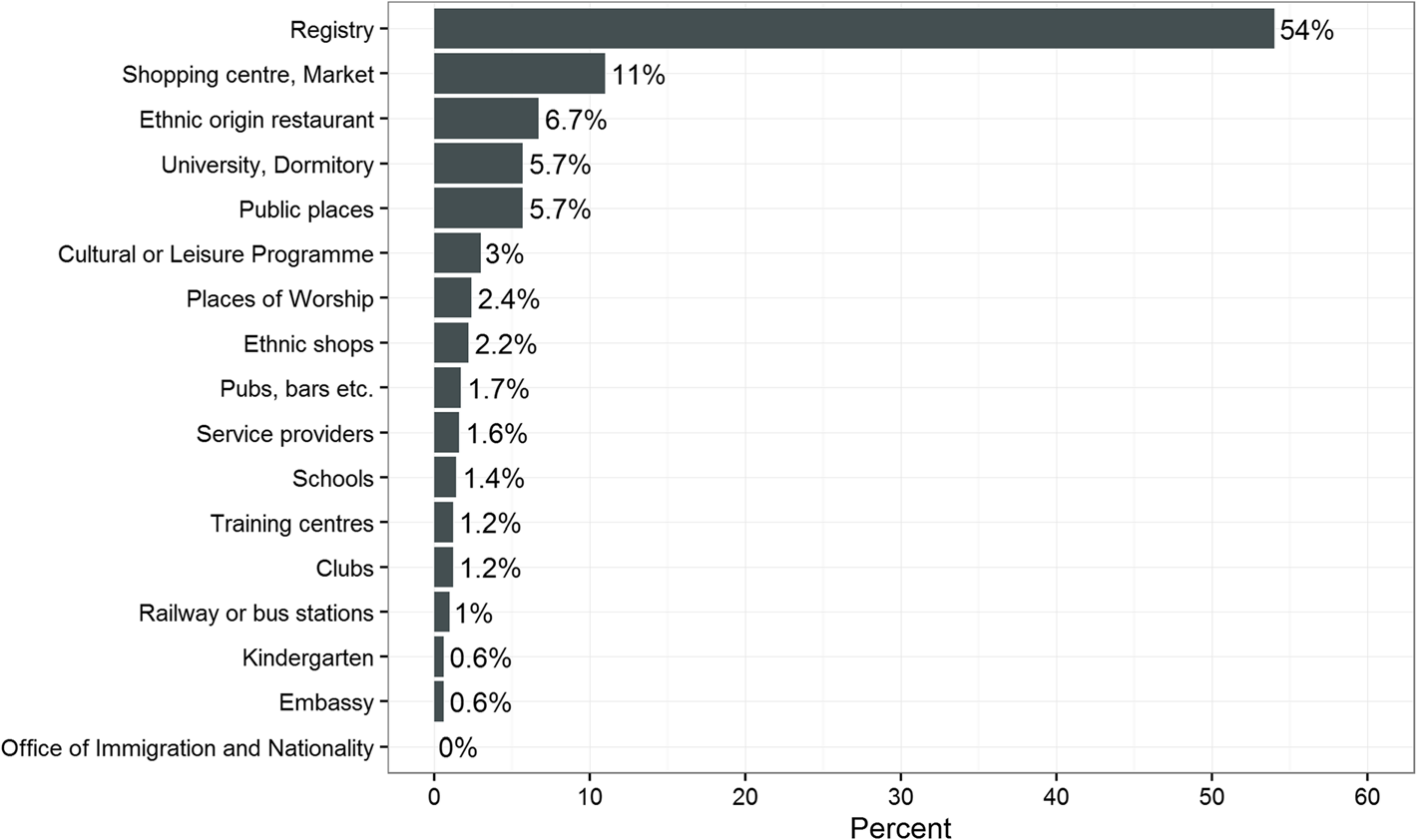
Centres of Aggregation and sampling intensity in Budapest by types of centres Source: Technical reports and information provided by Hungarian research team

Figure 2 shows all centre types covered in Hungary and the percentage of interviews sampled at each centre. In Budapest, 54% of all respondents interviewed came from the registry sample (interviews done at the addresses/homes of interviewees). The registry only includes naturalised immigrants, foreign citizens with long-term residence status and refugees. Therefore, location sampling was needed as a complement for coverage of the full range of TCFBs. Important locations were shopping centres and markets, ethnic restaurants, university and dormitories as well as public places. It was also planned to sample at the Office of Immigration and Nationality, however, approval for being allowed to sample at the office was received too late. Another difficulty for location sampling in Hungary and Portugal was that it was carried out for the first time and there was no experience on the likely distribution of the target population across the selected centres, which had to be assessed *ex ante* to some extent (information provided by Hungarian team via email).

**Fig. 3:**
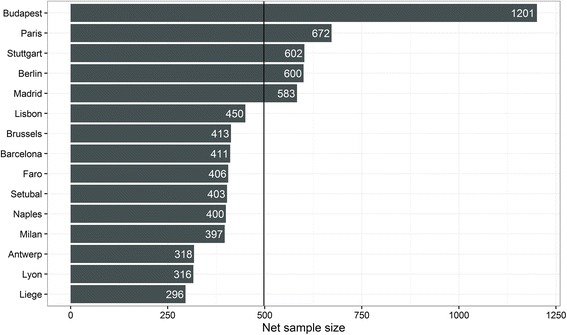
Sample sizes achieved in each city of the ICS (the *vertical line* gives the mean of 498)

Figure 3 shows the sample sizes achieved in all cities covered in the ICS. Altogether, 7473 persons were interviewed in the 15 cities. The minimum sample size was 300, which was missed by few interviews in one city. The samples in the cities ranged from 296 in Liege to 1201 in Budapest. The average sample size was 498. The main challenges of obtaining those samples of the target population with the methods outlined above are discussed in the next section.

### Main challenges and response rates

The study was carried out in 18 months including questionnaire design, translation, fieldwork and analysis. The fieldwork was undertaken by polling institutes in cooperation with national research partners in each of the countries. Carrying out the entire project within 18 months meant that researchers and polling institutes had to work under a considerable time pressure. While there was central coordination about key strategic questions, national researchers coordinated with national polling institutes in a somewhat decentralised fashion.

One key challenge arose from translating the questionnaire into different languages – which was done centrally, with validation checks by native speakers, who worked on the project – and particularly translating harmonised questions about residence status and residence permits. The central coordinating team tried to find proper translations, by adapting questions about residence status to each country’s specificities, but problems during fieldwork were reported about difficulties to understand those questions. This was, however, partly also explained by the fact that respondents did not have enough knowledge about their exact legal status, predominantly upon arrival in the country.

Response rates can to some extent indicate issues related to the quality of data obtained through fieldwork. Especially in surveys with immigrants and ethnic minorities, response rates might be influenced by additional factors, such as issues related to language skills, trust in the anonymity of the responses to the survey as well as non-contact due to the higher mobility of migrants (see for an overview Morales & Ros, 2013 or Kappelhof, 2015).

Information on response and refusals rates was not available in a standardised manner for the ICS and can only be reported for some countries. In surveys that include screening for particular rare target populations, such as the ICS, there are many invalid addresses due to ineligible addresses to be screened out. For instance, in Belgium 8378 addresses were approached. Almost half of these addresses were invalid (4085), because the addresses were not found, they were non-residential, inaccessible or – most notably – there was no eligible individual in the household.^7^ Of the 4293 valid addresses 1533 were unproductive because the interviewer was unable to contact anyone at the household (35.7%). Altogether 2760 effective contacts resulted in 1028 interviews, which results in a response rate after contact of 37% (therefore 63% refused). The response rates were similar across the three cities (Ipsos, 2012, p. 4). In France the success rate was even lower even though applied in areas with higher densities, as only 16% of screened persons resulted in an interview (as mentioned above). This might also be related to the fact that interviews were conducted over the phone, which usually results in higher refusals. Portugal was the only country applying location sampling that provided information on response rates. Altogether there were 1421 rejections, which results in a refusal rate of 53%. Higher rates of refusals were reported in residential areas and public spaces.

When compared to the response rates of surveys to the general population, the rates of the ICS remain rather low. For example, the European Social Survey (ESS) – targeting the general population – aims at reaching a 70% response rate, which is however not reached in several countries and response rates vary considerably. In recent rounds the ESS reached lower response rates of 35% in Germany and a rate just below 50% in France. Higher rates were obtained in Belgium with approx. 60%, in Hungary with 65%, in Spain with 70% and Portugal with 75% (Beullens, Matsuo, Loosveldt, & Vandenplas, 2014). However, it remains difficult to fully assess response rates for surveys such as the ICS due to the crucial importance of establishing eligibility of the net sample. In the case of non-contact and upfront refusal, there remains a large percentage of unknown eligibility. Assessing actual response rates remains challenging in the absence of this information (Smith, 2009), because it is not known to what extent non-contact or refusal to answer the screening questions differs among the general population and minorities.

One additional reason for lower response rates among immigrants is language problems. This problem is often mitigated by using bilingual interviewers and translated questionnaires. Yet, this problem is not as significant as might be assumed in most countries. There is information on response rates in the UK Labour Force Survey showing that language nonresponse is a factor hindering participation in the LFS. However, of all refusals to participate in the LFS, only 3.7% of all migrants sampled for the LFS 2008 in the UK did not respond because of language problems (Thomas, 2008, p. 42).^8^

The German and Spanish samples of the ICS also collected information on reasons for nonresponse. Interviews in Germany were done through computer assisted personal interviewing (CAPI), but paper questionnaires as supporting documents were available in other languages as well, including Albanian, Bosnian/Croatian/Serbian, English, French, Italian, Russian, Spanish and Turkish. Out of 4329 valid addresses in the German samples (in Stuttgart and Berlin), for 5.2% an interview was not possible due to language problems. The most important reasons for non-response were upfront refusal by the household or interviewee as well as eligible individuals not being located at their address.^9^ The situation was very similar in the Spanish cities (where translated questionnaires were also available in a number of languages), as the most important reason for non-response was unavailability of the respondent (30–34%) – the respondent was not at home every time the household was visited, or was not able to attend the interviewer at that time – and refusals (6–8%) or language problems (1–2%) were not very common.

**Fig. 4:**
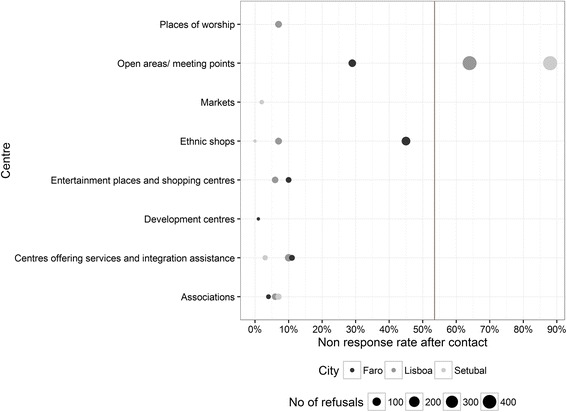
Non-response (refusal) rates obtained in Portuguese cities by centres (the *vertical line* gives the weighted mean of 53.5%) Source: ICS Technical report for Portugal

Response rates is a major issue with location sampling since it involves sampling and interviewing at unusual places. Information on response rates was provided by the Portuguese research team only. Figure 4 shows the response rates after contact (refusal rates) by city and centre in Portugal. These refusal rates need to be interpreted slightly differently compared to “traditional” response rates, since they reflect refusals after persons agreed to listen to what the interviewer proposes and if the person fits the target population. The overall refusal rate was 53%. Open areas and meeting points were most often used as centres (eight in Setubal, seven in Lisbon and four in Faro). Not surprisingly, refusal rates are much higher at public places, ranging from 29% in Faro to over 64% in Lisbon and to 88% in Setubal. With the exception of a high refusal rate in ethnic shops in Faro at 45%, all other centres report lower refusal rates at 11% or below. In the end, however, most interviews were obtained at public places. The situational aspect of willingness to participate in a survey is of particular importance for location sampling, such that we find locations with relatively high and relatively low refusal rates even across the same ‘type’ of centres/locations (e.g. open areas/meeting points or ethnic shops).

### Comparing the results of the ICS survey to other data sources

This section evaluates the quality of the ICS sampling approaches by comparing basic results on demographic variables in the samples of the ICS with data obtained on the same regional areas taken from the 2011 Census of the respective countries. Our analysis focuses on the data obtained in the countries where an adequate sampling frame was not available: the Belgian samples (random walk, partly with focused enumeration), the French samples (telephone screening in selected neighbourhoods) and the three samples in Hungary, Italy and Portugal based on location sampling. The comparisons are limited to the data availability for the regions in which the cities covered are located, as taken from the Eurostat 2011 Census Hub of the European Statistical System.^10^

The data provided through the Census Hub were not equally available for all the cities and the variation in the comparisons reflect data availability from the Census Hub. The comparisons include age groups, gender, period of immigration, nationality and education. The estimates provided for the ICS include 95% confidence intervals as estimated from the data when the sampling weights provided in the dataset are applied. Since confidence interval estimation from more complex samples is not always straightforward, the intervals shown only provide an indication of the potential sampling error. The data from the Census Hub include all persons born outside the current EU, which means that persons born abroad as nationals of the survey countries are included in the census data as well. This might lead to differences in the samples for Portugal and France, where there is a considerable share of people born in former colonies to Portuguese and French emigrants as well as a considerable proportion of returned children of expatriates/emigrants. It is important to mention that such data were not (easily) available at the time the survey was designed and first results analysed. These comparisons were only possible at a later stage after the census data – collected at the same time in most countries – became available.

#### Age

**Fig. 5:**
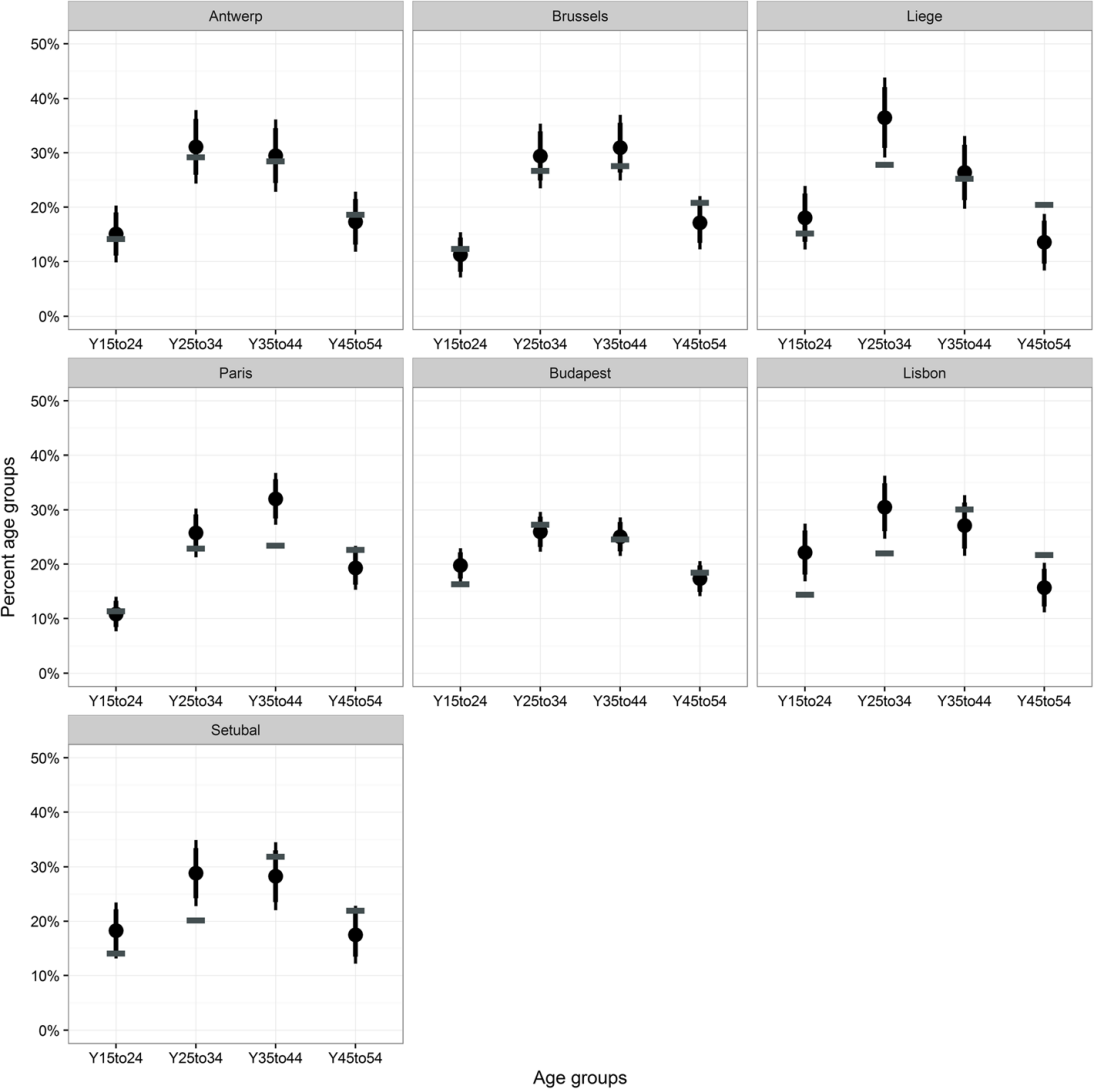
Comparison of age groups in the ICS sample (*solid circle* for estimate and 95% confidence interval bars) with census data (*horizontal bar*)

As a starting point, the distributions of the different age groups were compared. The age groups were selected based on the availability of age groups in both datasets. The comparison (Fig. 5) shows a slight under-estimation of the ‘older’ age groups in almost all ICS samples and a tendency of over-representation of the age groups 25–34. Although the distributions are comparable, the samples in Liege, Lisbon and Setubal show a clear over-representation of the age group 25–34 and an under-representation of the group at ages 45–54. In the sample in Paris the group of 35–44 years old is significantly over-represented. The over-representation of younger members of the target group is to be expected in location sampling, as the older age groups will be less likely to be out and about in public places. The over-representation of the middle-aged group in French cities is likely to be a consequence of differential telephone line subscription.

#### Gender

**Fig. 6:**
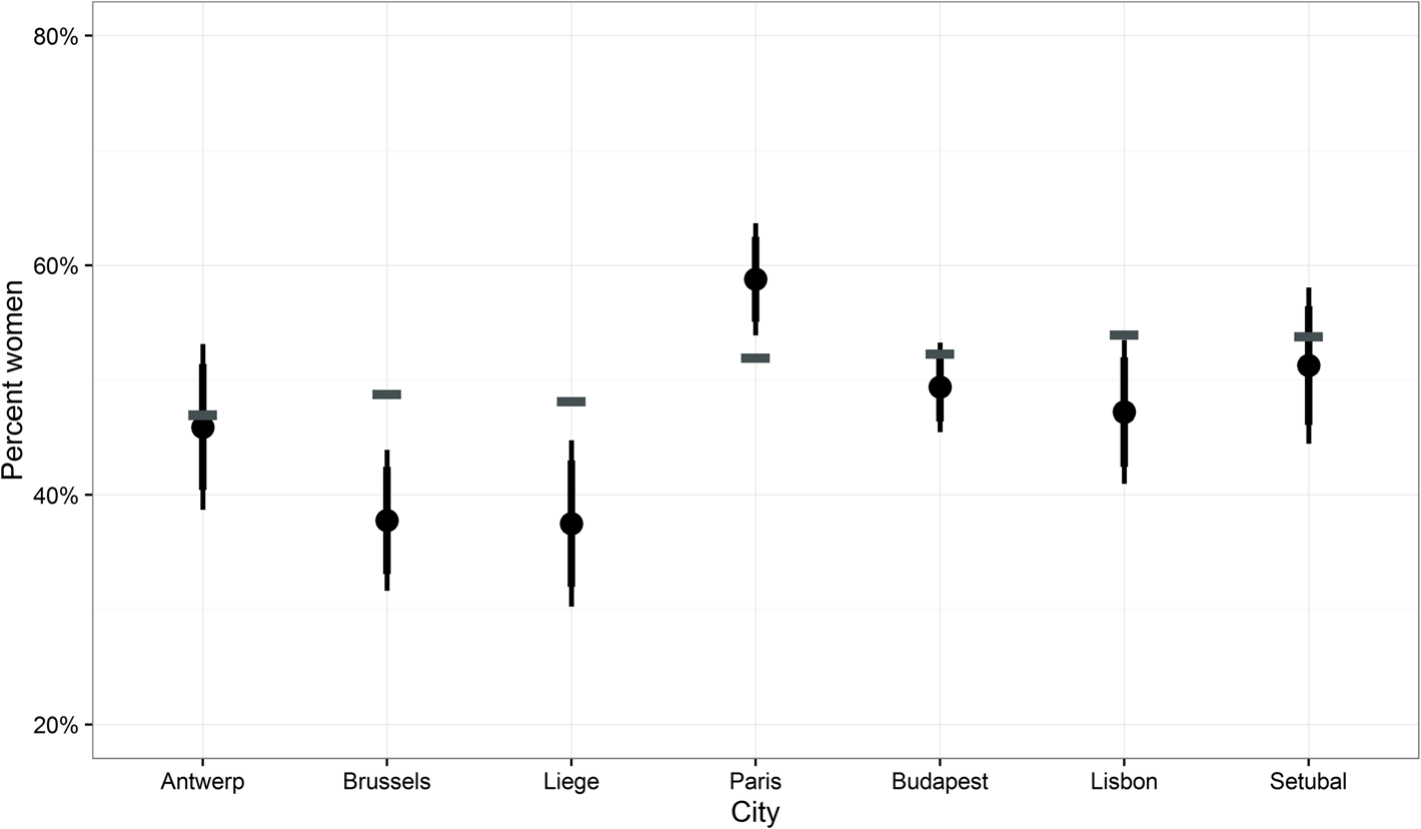
Comparison of gender distribution in the ICS sample with census data

As compared to the census data, the samples in Brussels and Liege as well as in Lisbon clearly under-represent women, while the sample in Paris clearly over-estimates the percentage of women (Fig. 6). The other samples come quite close to the gender distribution enumerated in the census 2011. The fact that the ICS sample in Antwerp correctly estimates the gender distribution, while those in Brussels and Liege depart significantly points to likely reasons different to sampling strategy for these departures, such as differential contact and response rates that might be driven by differences in the compositions of the immigrant population across different localities of the same country. Equally, with the same sampling methods, the Portuguese sample in Setubal approximates the census figures while it departs in Lisbon, thus providing additional indication that sampling design is not necessarily the key factor in determining the correct representation of underlying populations.

#### Year of immigration

**Fig. 7:**
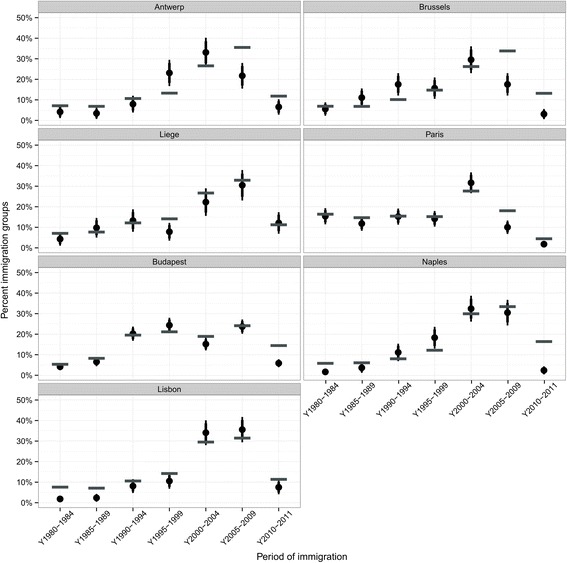
Comparison of distribution of periods of immigration in the ICS sample with census data

The period of immigration is an important key characteristic of immigrants’ life experiences, since the length of residence is an important factor impacting on the level of integration of immigrants and indicating different waves and conditions of immigration, which have been historically different over decades in the countries. In most cities included in Fig. 7, with the exception of Liege, the most recent years (2010 and 2011) tend to be under-represented. This is not surprising because recent immigrants are more difficult to capture in surveys due to several reasons, such as higher tendencies of having language difficulties, lack of trust in their new countries of residence, high mobility or the tendency to live in institutional households, which are often not covered in sampling frames. In Brussels, Antwerp and Paris immigrants arriving between 2005 and 2009 are also considerably under-represented. The three cities applying location sampling included in Fig. 7 fare quite well with respect to the distribution by year of immigration. In Lisbon (here Greater Lisbon, which combines the two cities of Lisbon and Setubal), there is a slight over-representation of immigrants arriving between 2000 and 2009 at the expense of a slight under-representation of immigrants coming in the 1980s.

#### Nationality

**Fig. 8:**
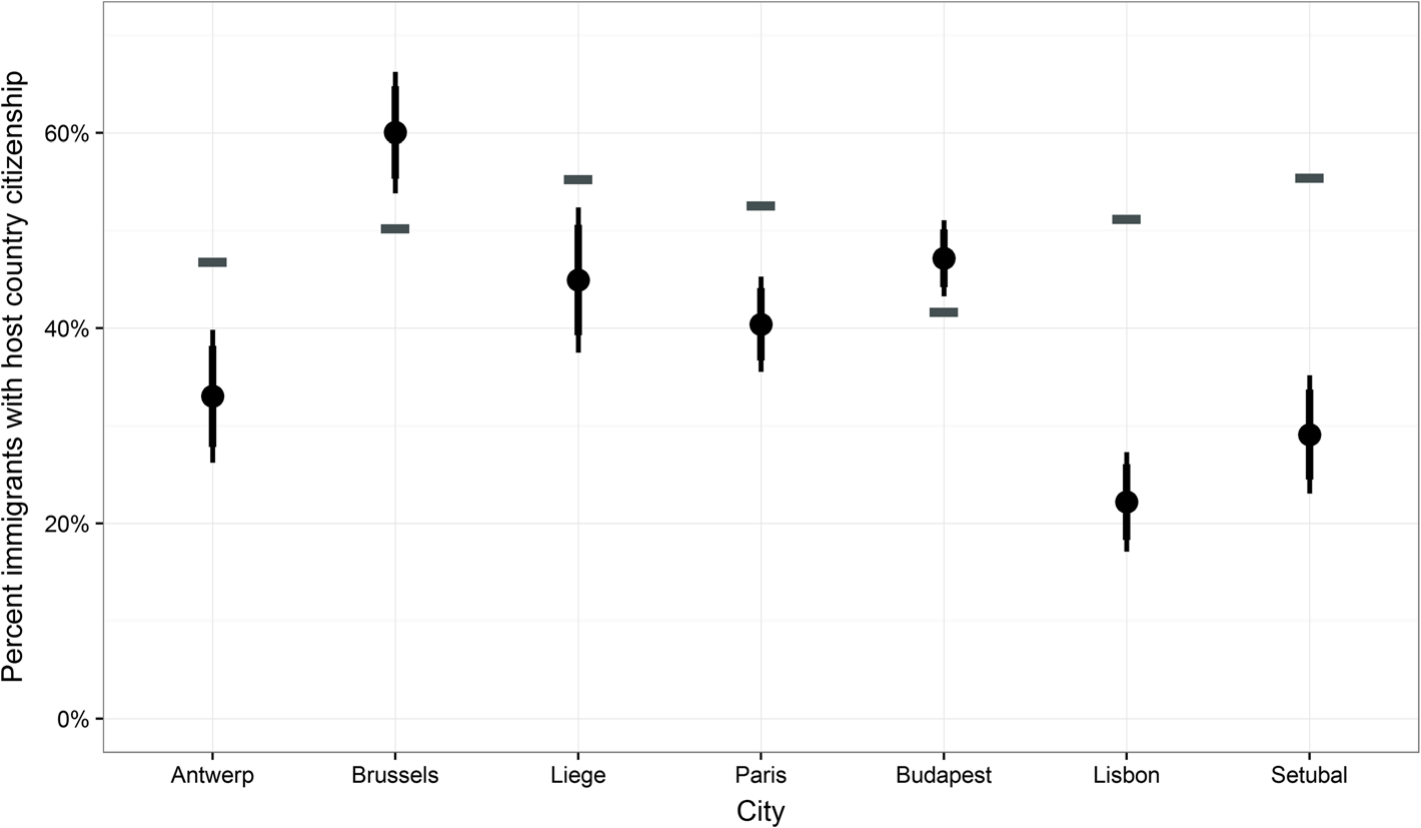
Comparison of percentages of target group with nationality of country of residence

Looking at the samples by nationality, the percentage of nationals among immigrants differs quite strongly in all cities. As mentioned above, the case of Portugal might be different due to children of Portuguese emigrants who returned to Portugal being included in the census but not in the sample of the ICS. This is indicated by the strong differences in the rates of nationals, which is considerably lower in the ICS. The same is true for the sample of Paris, Liege and Antwerp. Contrary to that, there is an over-representation of nationals in the ICS in Brussels and, to a lesser extent, in Budapest. Yet, what the results in Fig. 8 suggest is that there is no intrinsic bias in a given direction for any of the specific sampling methods. With the same sampling methods, some Belgian samples over-estimate the percentage of naturalised immigrants while others underestimate it. Equally, with location sampling, the estimate for Budapest is not too far removed from the census figure, whereas the departure of the Portuguese estimates is very considerable. The reasons for this disparity can be multiple. It could be due to some form of under-estimation of the underlying real value in the population, but it might also be related to the differences between the two populations considered, as the census also includes immigrants from former colonies born to Portuguese emigrants in the colonies, who were not the target of the ICS.

#### Education

**Fig. 9:**
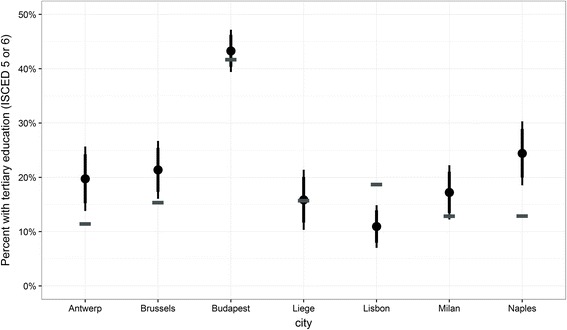
Comparison of percentages of the target group with tertiary education

Finally, the percentage of respondents with tertiary education in the ICS is compared to the percentage reported in the census (Fig. 9). While the CAT sample in Budapest and the random walk plus focused enumeration in Liege match the census data quite well, the location sample in Milan and Naples as well as the random walk samples in Antwerp and Brussels over-represent highly educated immigrants. In Greater Lisbon, the level of education in the ICS is lower than according to the census.

The comparison thus shows that the ICS samples that did not employ population registers as the sampling frame fare unevenly in their ability to produce representative samples. While they perform reasonably well for some characteristics (age and period of immigration), they perform poorly for others (nationality) and variably yet for others (gender and education). The comparisons provided also suggest that there is no clear ‘winner’ or ‘loser’ in terms of the capacity to render representative samples of the various sampling methods considered. With the same sampling methods, the accuracy of the estimates for the Belgian cities varies in magnitude and direction. Equally, with very similar sampling designs, the Budapest estimates seem to outperform those of the Italian and Portuguese cities on most variables considered. With the data at hand, we cannot assess to what extent the differences in sample and census estimates stem from the sampling approaches or their fieldwork application, from the disparities in the definition of the target group in the ICS samples and in the census data, or from non-response. Most likely, they are the result of the combined effect of these three aspects.

## Discussion and conclusions

The lack of adequate sampling frames for immigrants and ethnic minorities makes it important to further develop alternative field methods that can achieve – or approach – probability samples. This paper provided an overview of the results and experience gathered through a specialised survey carried out to TCFB people in selected European cities. The ICS provides a valuable addition both to the substantive knowledge of immigrant populations across Europe and to the development of methodological tools that will contribute to making further progress in the study of hard-to-reach populations.

Despite the challenges of the implementation of such a survey, including low response rates and implementing new and not very well tested methods, the results show that the survey could deliver meaningful results. Although there are some deviations in comparison to the census data, the samples match the distribution with respect to some key characteristics – most notably periods of immigration.

The paper highlights that traditional methods – such as random walk sampling or telephone sampling – are also limited in providing results for the target population, due to the necessary exclusion of a certain part of the target population (i.e. lower density areas due to too high screening costs) and potentially other limitations (e.g. high refusal rates). We found that alternative methods such as location sampling can deliver even more accurate results as compared to the total target population. Particularly the results of the sample in Budapest, which are based on a mix of register-based sampling and location sampling, are promising and point to the importance of further investigation into alternative sampling designs that combine multiple strategies in creative ways. Location sampling in combination with (imperfect) register sampling (such as residence permit data) seem to be a reasonable approach for making up deficits in existing registers.

Based on the results of this analysis, the following recommendations for future surveys can be made. Traditional sampling methods (e.g. multi-stage sampling with screening for the target population) are generally preferable due to their known characteristics and wider application. However, for minor domains (sub-populations), such as immigrants, the coverage of the survey usually needs to be limited through either excluding certain areas with lower concentration of the target population and/or further geographical restrictions to make the survey feasible with limited resources (for example by only covering selected cities in a country). This, however, excludes a certain part of the target population from the survey by definition. A restriction of the survey target group to cities, for example, is a common approach because nation-wide samples can be rarely financed. Such limitations need to be discussed and taken into account when the results are presented and interpreted. As discussed with respect to the interpretation of general migrant integration indicators, the contextualisation of results is crucial for the interpretation of statistics on migrants, particularly on the international level (see for example the discussion in Huddleston, Niessen, & Tjaden, 2013). Close scrutiny is necessary when comparing cities across and within a country to understand differences reported in a survey. As an example, the ICS yields different results for the cities covered in Belgium. These come from differences in the situation of the target population in the cities. The contextual situation in terms of immigrant integration differs across the country, given the different immigration waves and trajectories experienced in Wallonia (Liège) and Flanders (Brussels and Antwerp), as well as the particular situation for Brussels in certain aspects, especially as a magnet of highly-qualified professionals not just as the Belgian capital city but also the administrative capital of European institutions (cf. for example Phalet & Swyngedouw, 2003).

When traditional methods are not deemed feasible, alternative methods, such as network or location sampling, need to be employed. While location sampling seems superior in most cases, the decision of which method to use depends on the purpose of the survey and resources available. For all alternative methods – as well as traditional ones – thorough knowledge about the target population needs to be collected in advance of the fieldwork in order to properly carry out the survey. The collection of data about the target population not only helps to design and contextualise a survey, but also allows improving the survey outcomes through non-response adjustments and post-stratification. In the specific case of ICS, the opportunity to improve the survey design through detailed pre-fieldwork statistical analysis was not (easily) available at the time of the survey, because (recent) relevant census data were not available in most countries in 2011. The availability of data and opportunities for secondary data analysis has considerably increased over the past decade, which creates an important source of information for improving and contextualising survey data. Despite all the challenges and costs related to collecting high quality survey data, the collection of such data becomes ever more important in times where migration (related) policy will remain prominent on the policy agenda.

Survey data remain an indispensable source of information, because only survey data can deliver certain types of information that non-survey data (e.g. data collected from people’s behaviour on the internet) cannot, such as people’s experiences and opinions, in a way that we can safely make inferences to the larger target population. It is important to highlight that the quality of data can only be assessed when full transparency of the data collection process is published alongside the results of a survey – or of any type of empirical data collection.

## Footnotes

1
www.mipex.eu, accessed on 14 April 2016.

2
http://fra.europa.eu/en/project/2015/eu-midis-ii-european-union-minorities-and-discrimination-survey, accessed on 14 April 2016.

3 Comprehensive overviews of sampling methods for specific sub-populations can be found in Sudman and Kalton (1986), Kalton (2009), Marpsat and Razafindratsima (2010) or Verma (2013).

4 The authors refer to it as the 'intercept method'.

5 By contrast, TCNs could include the children of immigrants who did not obtain Belgian citizenship. However, this number is assumed to be low, especially for the Belgian case due to favourable nationality acquisition regulations that are, in practice, close to *jus soli*.

6 There is evidence that using different modes of interviews has some implications concerning measurement effects. Telephone interviews are not as much affected by social desirability as compared to face-to-face interviews (see e.g. Vannieuwenhuyze, Loosveldt, & Molenberghs, 2010). At the same time, evidence suggests that “satisficing” (i.e. giving indifferent responses) might be higher in telephone surveys (Martin, 2011).

7 A minor number also dropped out because of health or language problems.

8 The main reasons for non-response in this study were broken appointments, slightly higher among immigrants (47.1% compared to 44.6% non-migrants) and the excuse of being too busy to take part in the survey (15% migrants and 13.9% non-migrants). Distrust in anonymity is also higher among immigrants, but it only makes up a very small share of non-response, below 1% (Thomas, 2008).

9 Information provided by country partner, based on background analysis from the polling institute IFAK.

10 Available at: Eurostat 2011 Census Hub https://ec.europa.eu/CensusHub2/ (last accessed in August 2015)

## References

[CR1] Baio G, Blangiardo GC, Blangiardo M (2011). Centre sampling technique in foreign migration surveys: a methodological note. Journal of Official Statistics.

[CR2] Beauchemin C, González-Ferrer A (2011). Sampling international migrants with origin-based snowballing method: New evidence on biases and limitations. Demographic Research.

[CR3] Beauchemin, C., Hamel C., & Simon P. (2016). Trajectoires et origines. Enquête sur la diversité des populations en France [Trajectories and origins. Survey of Population Diversity in France]. INED

[CR4] Berry B (2007). A repeated observation approach for estimating the street homeless population. Evaluation Review.

[CR5] Beullens K, Matsuo H, Loosveldt G, Vandenplas C (2014). Quality report for the European Social Survey, round 6.

[CR6] Blangiardo, G. C. (2008). *The centre sampling technique in surveys on foreign migrants. The balance of a multi-year experience*. Eurostat Working Paper 12, 29 February 2008, http://www.unece.org/fileadmin/DAM/stats/documents/ece/ces/ge.10/2008/wp.12.e.pdf, Accessed 28 May 2014.

[CR7] Braun M, Santacreu O, Recchi E, Favell A (2009). Appendix A. Methodological notes. Pioneers of European integration. Citizenship and mobility in the EU.

[CR8] Brouard S, Tiberj V (2011). As French as everyone else? A survey of French citizens of Maghrebin, African, and Turkish origin.

[CR9] Crul M, Schneider J, Lelie F (2012). The European second generation compared. Does the integration context matter?.

[CR10] Duque I, Ballano C, Perez C, Font J, Mendez M (2013). The 2007 Spanish National Immigrant Survey (ENI): Sampling from the Padrón. Surveying ethnic minorities and immigrant populations: Methodological challenges and research strategies.

[CR11] Erens B, Font J, Mendez M (2013). Designing high-quality surveys of ethnic minority groups in the United Kingdom. Surveying ethnic minorities and immigrant populations: Methodological challenges and research strategies.

[CR12] Ersanilli E, Koopmans R (2011). Do immigrant integration policies matter? A three-country comparison among Turkish immigrants. West European Politics.

[CR13] European Union Agency for Fundamental Rights (FRA) (2009a). EU-MIDIS. Main results report, http://fra.europa.eu/en/publication/2012/eu-midis-main-results-report, Accessed on 14 Apr 2016.

[CR14] European Union Agency for Fundamental Rights (FRA) (2009b). EU-MIDIS technical report. Methodology, sampling and fieldwork, http://fra.europa.eu/sites/default/files/eu-midis_technical_report.pdf, Accessed on 24 Mar 2016

[CR15] Eurostat (2011). Indicators of immigrant integration. A pilot study.

[CR16] Font, J., & Mendez, M. (Eds.). (2013). *Surveying ethnic minorities and immigrant populations: Methodological challenges and research strategies*. Amsterdam: IMISCOE Research, Amsterdam University Publications.

[CR17] Gile K, Johnston L, Salganik M (2015). Diagnostics for respondent-driven sampling. Journal of the Royal Statistical Society: Series A (Statistics in Society).

[CR18] Goodman LA (1961). Snowball sampling. The Annals of Mathematical Statistics.

[CR19] Heckathorn DD (1997). Respondent-driven sampling: A new approach to the study of hidden populations. Social Problems.

[CR20] Huddleston T, Niessen J, Chaoimh EN, White E (2011). Migrant integration policy index III.

[CR21] Huddleston T, Niessen J, Tjaden JD (2013). Using EU indicators of immigrant integration.

[CR22] Huddleston, T., & Tjaden J. T. (2012) *Immigrant citizens survey. How immigrants experience integration in 15 European cities*. Brussels: King Baudouin Foundation and Migration Policy Group, www.immigrantsurvey.org, Accessed 19 Feb 2016.

[CR23] Ipsos (2012). Immigrant citizens survey. Technical report, http://www.immigrantsurvey.org/downloads/ICS%20-%20technical%20report%20-%20Belgium.pdf, Accessed 12 Aug 2015

[CR24] Ipsos MORI & TNS-BMRB (2010). 2010-11 Citizenship survey technical report, London, http://doc.ukdataservice.ac.uk/doc/7111/mrdoc/pdf/7111_technical_report.pdf, Accessed 6 Sept 2015

[CR25] Kalton G (2009). Methods for oversampling rare subpopulations in social surveys. Survey methodology.

[CR26] Kappelhof, J. (2015). *Surveying ethnic minorities: The impact of survey design on data quality* (Doctoral dissertation). Published on the website of the Netherlands Institute for Social Research, University of Utrecht. Retrieved from http://www.scp.nl/Publicaties/Alle_publicaties/Publicaties_2015/Surveying_ethnic_minorities, Accessed 6 Sept 2015.

[CR27] Kish L (1987). Statistical Design for Research.

[CR28] Kraler A, Reichel D (2010). Statistics on migration, integration and discrimination in Europe. PROMINSTAT final report.

[CR29] Landry PF, Shen M (2005). Reaching migrants in survey research: The use of the global positioning system to reduce coverage bias in China. Political Analysis.

[CR30] Latcheva R, Lindo F, Machado F, Pötter U, Salentin K, Stichs A (2006). Immigrants and ethnic minorities in European cities: Life-courses and quality of life in a world of limitations.

[CR31] Latcheva, R., Reichel D., Till-Tentschert U. (2016): *Surveying hard-to-reach groups from a comparative cross-country perspective: The Second European Union Minorities and Discrimination Survey (EU-MIDIS II)*, UNECE Working Paper 25, Work Session on Migration Statistics, Geneva, http://www.unece.org/fileadmin/DAM/stats/documents/ece/ces/ge.10/2016/mtg2_WS/25_Latcheva_EU_FRA_final_revised.pdf, Accessed 2 Oct 2016

[CR32] Marpsat M, Razafindratsima N (2010). Survey methods for hard-to-reach populations: Introduction to the special issue. Methodological Innovations Online.

[CR33] Martin P (2011). A good mix? Mixed mode data collection and cross-national surveys. Ask Research & Methods.

[CR34] McKenzie D, Mistiaen J (2009). Surveying migrant households: A comparison of census- based, snowball, and intercept point surveys. Journal of the Royal Statistical Society.

[CR35] Morales L, Giugni M (2011). Social capital, political participation and migration in Europe. Migration, minorities and citizenship.

[CR36] Morales, L., & Ros, V. (2013). Comparing the response rates of autochthonous and migrant populations in nominal sampling surveys: The LOCALMULTIDEM study in Madrid. In J. Font & M. Mendez (Eds.), *Surveying ethnic minorities and immigrant populations: Methodological challenges and research strategies* (pp. 147–172). Amsterdam: IMISCOE Research, Amsterdam University Publications.

[CR37] Phalet K, Swyngedouw M (2003). Measuring immigrant integration: The case of Belgium. Studi Emigrazione.

[CR38] Recchi E, Favell A (2009). Pioneers of European integration. Citizenship and mobility in the EU.

[CR39] Schnell, R., Gramlich T., Bachteler T., Reiher J., Trappmann M., Smid M., …Becher I. (2013). *A new name-based sampling method for migrants using n-grams*. German Record Linkage Center – Working Paper Series NO. WP-GRLC-2013-04, http://www.record-linkage.de/-download=wp-grlc-2013-04.pdf Accessed 24 Mar 2016.

[CR40] Smith, T. W. (2009). *A revised review of methods to estimate the status of cases with unknown eligibility*. Available at the website of the American Association for Public Opinion Research (AAPOR): https://www.aapor.org/AAPOR_Main/media/MainSiteFiles/FindingE.pdf, Accessed 9 December 2016.

[CR41] Sudman S, Kalton G (1986). New developments in the sampling of special populations. Annual Review of Sociology.

[CR42] Thomas, M. (2008). *Improving migrant participation in the labour force survey: Non-response and attitudes of non-English speaking migrants to participation* (pp. 39–51). Survey Methodology Bulletin, No. 63, September 2008, Office for National Statistics

[CR43] Vannieuwenhuyze J, Loosveldt G, Molenberghs G (2010). A method for evaluating mode effects in mixed-mode surveys. Public Opinion Quarterly.

[CR44] Verma V (2013). Sampling elusive populations: Applications to studies of child labour.

[CR45] Williams M (2010). Can we measure homelessness? A critical evaluation of ‘Capture-Recapture’. Methodological Innovations Online.

